# Supplementary material for: Accessing the bottleneck in all-solid state batteries, lithium-ion transport over the solid-electrolyte-electrode interface
Source: Nat Commun. 2017 Oct 20;8:1086. doi: 10.1038/s41467-017-01187-y (PMC5651852; doi:10.1038/s41467-017-01187-y)
Supplement: Supplementary file 1 — Supplementary Information [file 41467_2017_1187_MOESM1_ESM.pdf]

Supplementary Figures

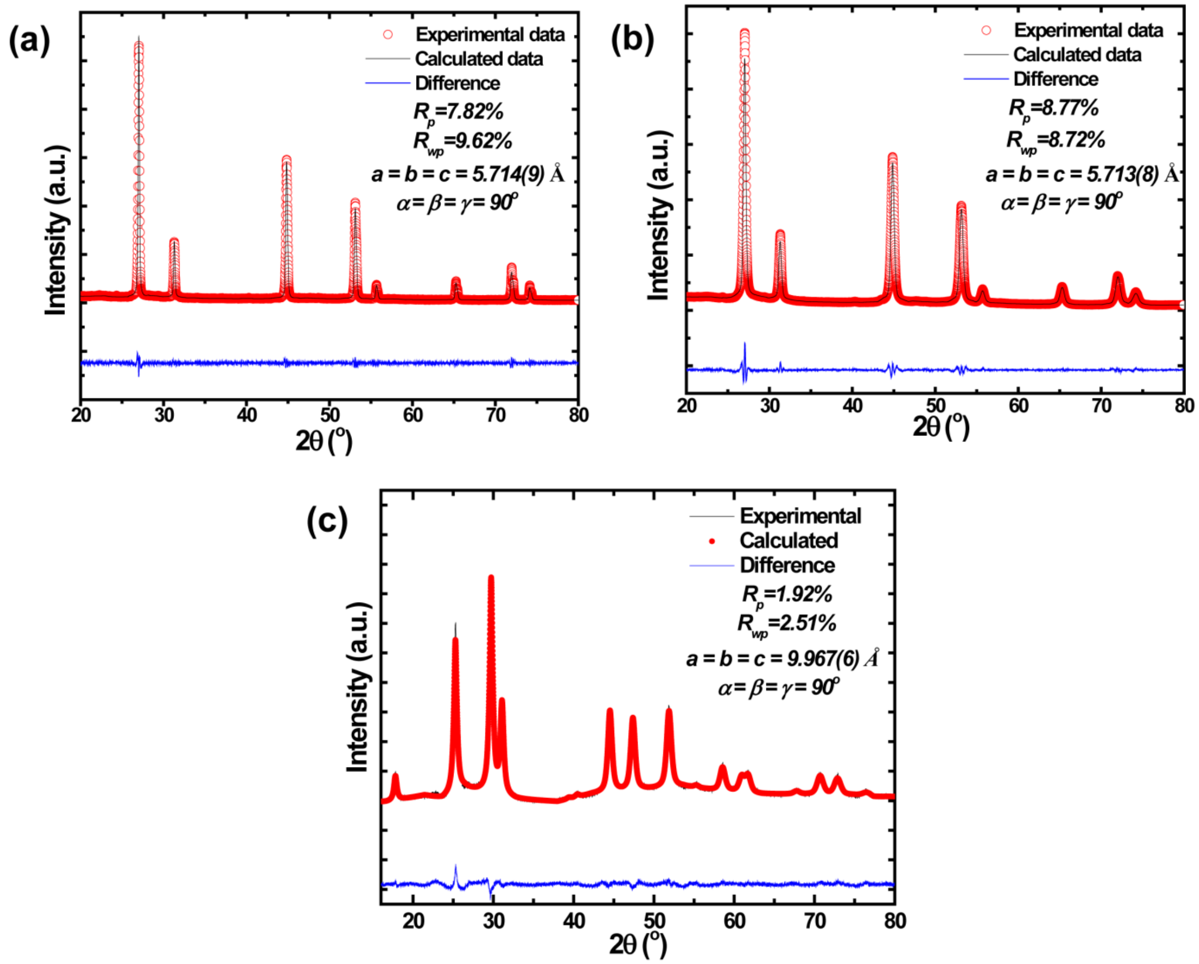

Supplementary Figure 1. Determination of average crystallite sizes. XRD and Rietveld refinement of commercial Li_2S (a), nano Li_2S (b), and the Li_6PS_5Br solid electrolyte (c). The average crystallite sizes are 120, 38, and 23 nm respectively, all of which are obtained based on the above refinement results.

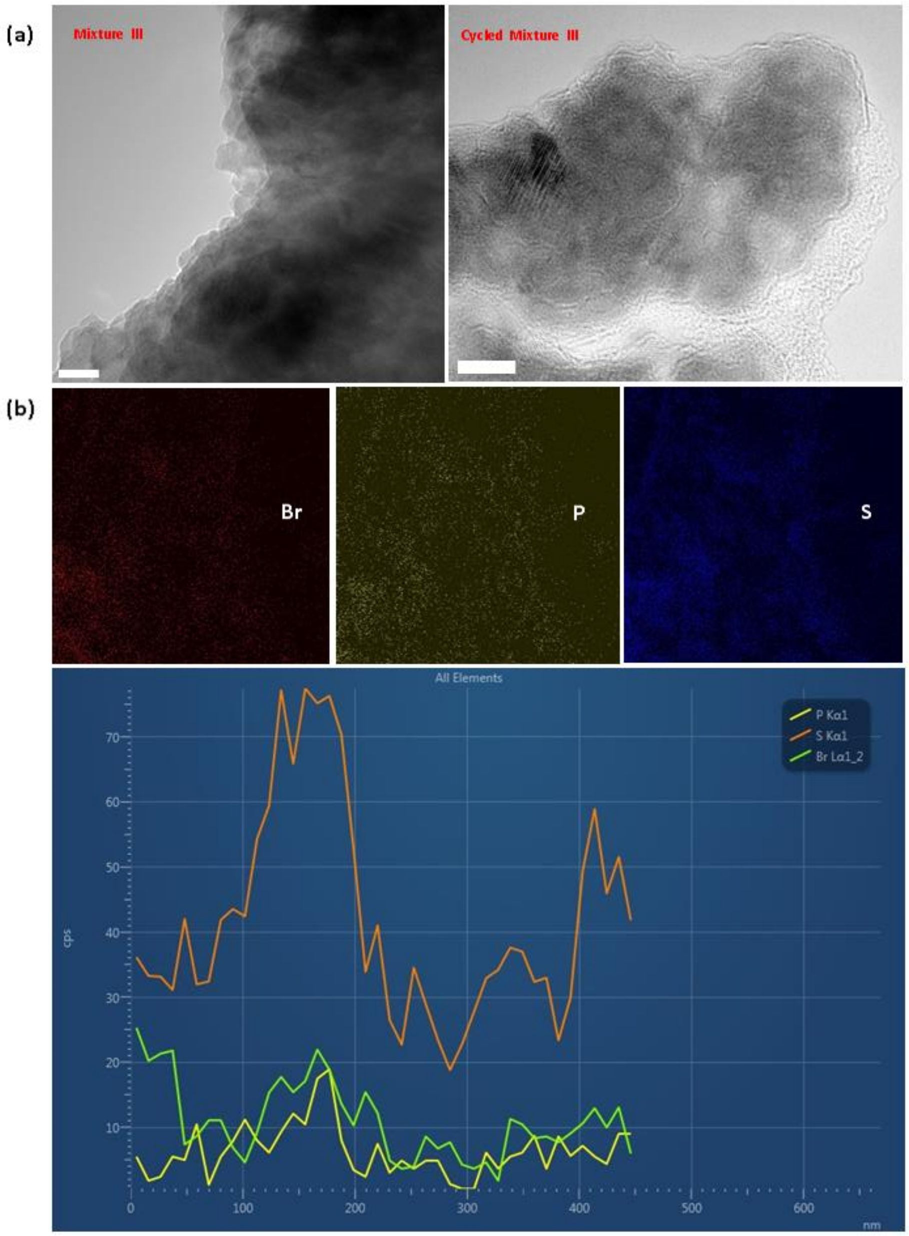

Supplementary Figure 2. Determination of crystallite size and elemental distribution. (a) TEM images of pristine and cycled mixture III. (b) EDX mapping of Br, P, S and a line map for mixture III. The EDX mapping, and line scan indicate that the mixing of the Li_2S and the Li_6PS_5Br is on a length scale smaller than 100 nm. The scale bar for the images in (a) corresponds to 10 nm.

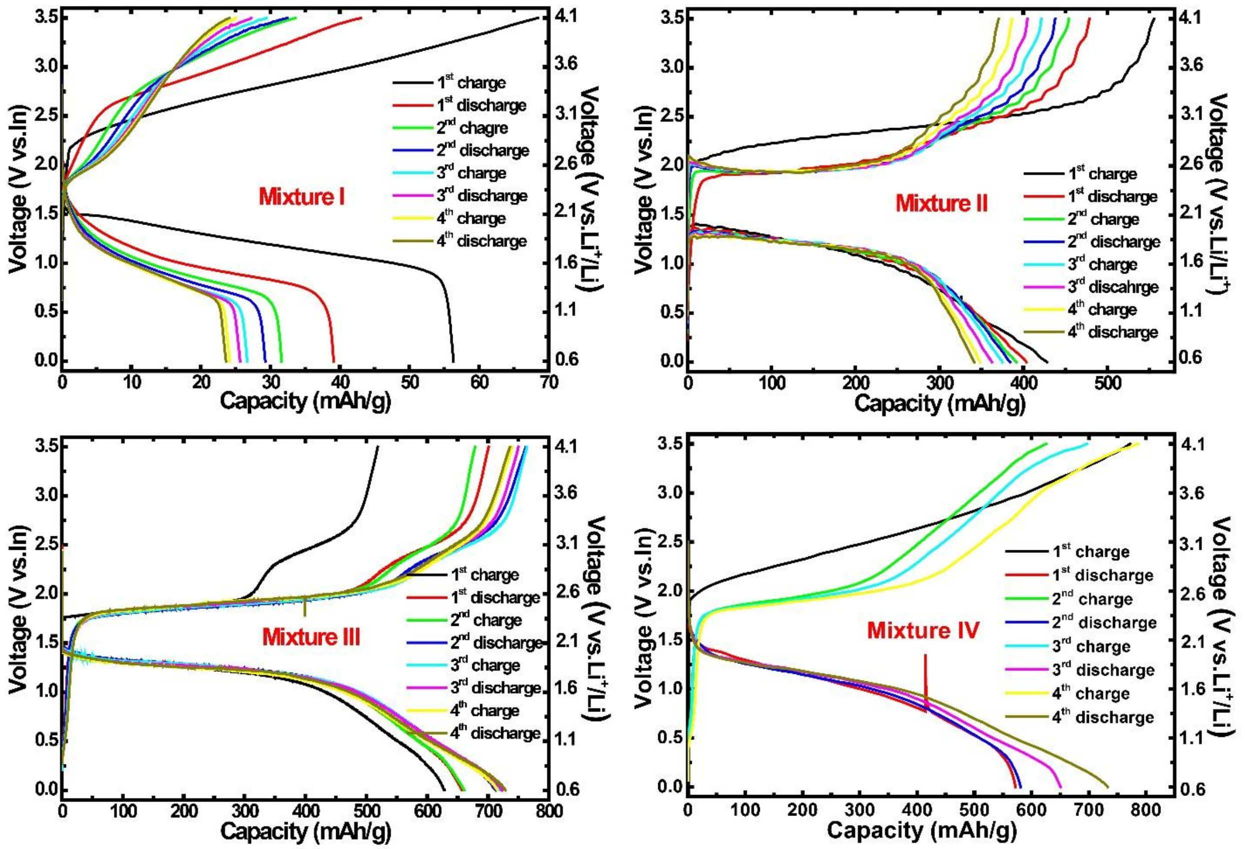

Supplementary Figure 3. Electrochemical voltage profiles of mixtures I-IV. Charge and discharge curves of cathodes comprising of mixture I (commercial Li_2S) mixture II (nano Li_2S), mixture III (ball milled nano Li_2S and Li_6PS_5Br), and mixture IV (annealed mixture III). The batteries were cycled versus an In foil anode at a current density of 0.064 mA/cm^2 in the voltage window of 0 - 3.5 V vs. In (0.62-4.12 V vs. Li^+/Li).

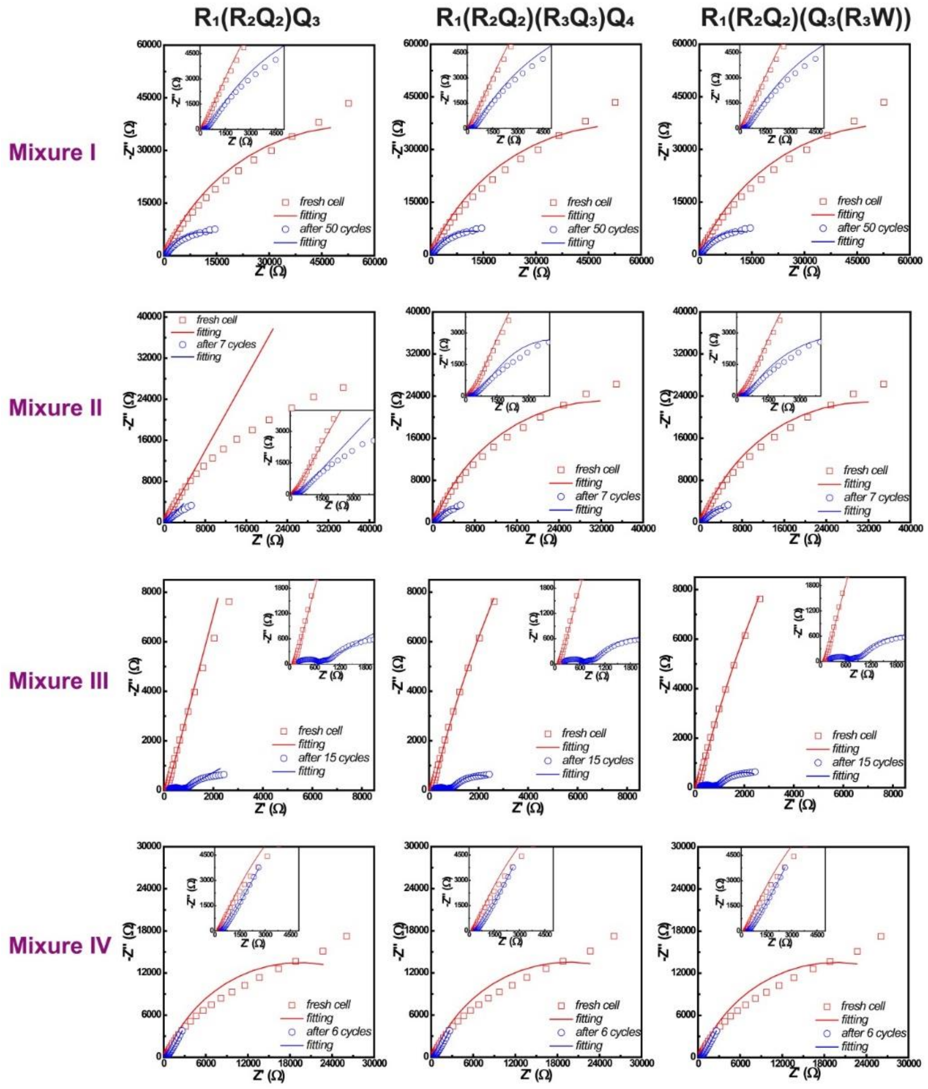

Supplementary Figure 4. Part I. EIS curves fit using three different equivalent circuits. EIS curves and fits of the fresh and cycled solid-state batteries using different Li_2S-Li_6PS_5Br mixtures (I-IV) mixed with carbon with a weight ratio of 2:2:1 as cathode combined with Li_6PS_5Br electrolyte and In foil anode. The resuts are fitted by $R_1(R_2Q_2)Q_3$, $R_1(R_2Q_2)(R_3Q_3)Q_4$ and $R_1(R_2Q_2)(Q_3(R_3W))$ equivalent circuits. For the equivalent circuit $R_1(R_2Q_2)Q_3$, R_1 reflects the resistance of the solid electrolyte, and R_2 reflects the Li_2S cathode electrode-Li_6PS_5Br solid electrolyte interface. The application of the two other equivalent circuits $R_1(R_2Q_2)(R_3Q_3)Q_4$ is motivated by the appearance of a new semicircle in the cycled data, indicating the formation of an additional interface upon cycling, possibly at the In anode.

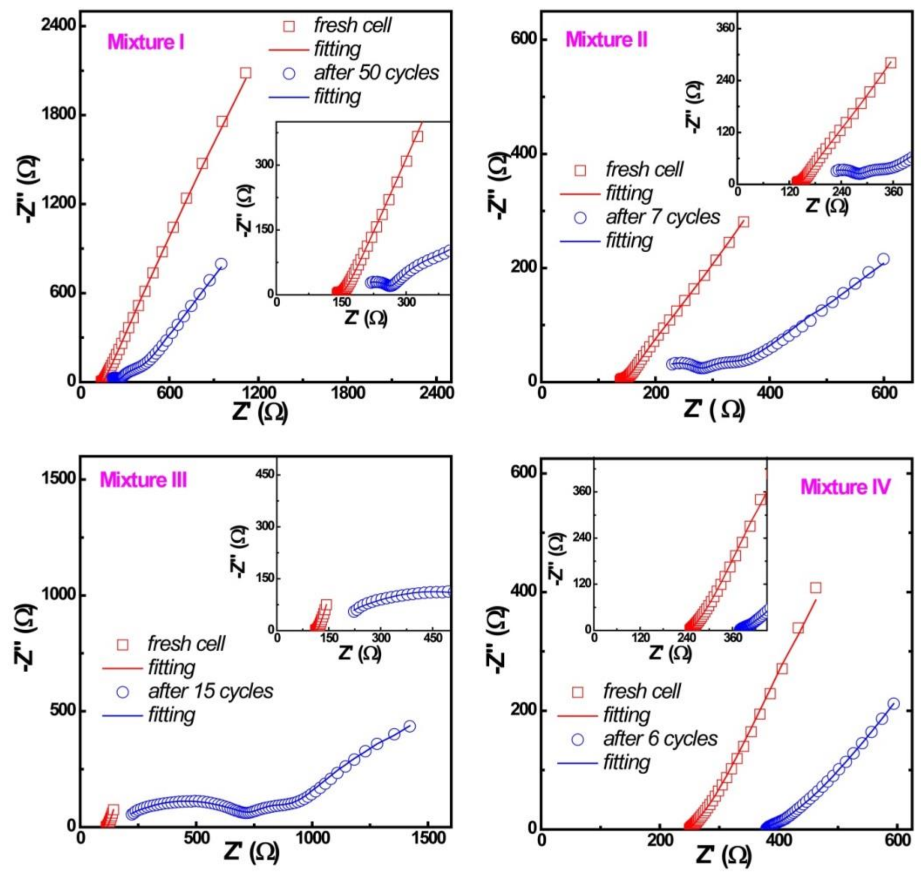

Supplementary Figure 4. Part II. EIS curves fit to get an accurate estimation of R_1. EIS curves of the fresh and cycled solid-state batteries using different Li_2S-Li_6PS_5Br mixtures (I-IV) mixed with carbon with a weight ratio of 2:2:1 as cathode combined with Li_6PS_5Br electrolyte and In foil anode. The EIS data of fresh cells are fitted by the equivalent circuit $R_1(R_2Q_2)Q_3$, while the data for cycled cells are fitted by the equivalent circuit $R_1(R_2Q_2)(R_3Q_3)Q_4$, the latter motivated by the appearance of an additional semicicle which indicate the formation of an additional interface upon cycling, possibly at the In anode. Because the aim is to get an accurate estimation of R_1 (the bulk solid electrolyte resistance) and R_2 (the resistance of the Li_2S-Li_6PS_5Br interface) for comparison with the NMR experiments only the high frequency part of the EIS data was included in the fit.

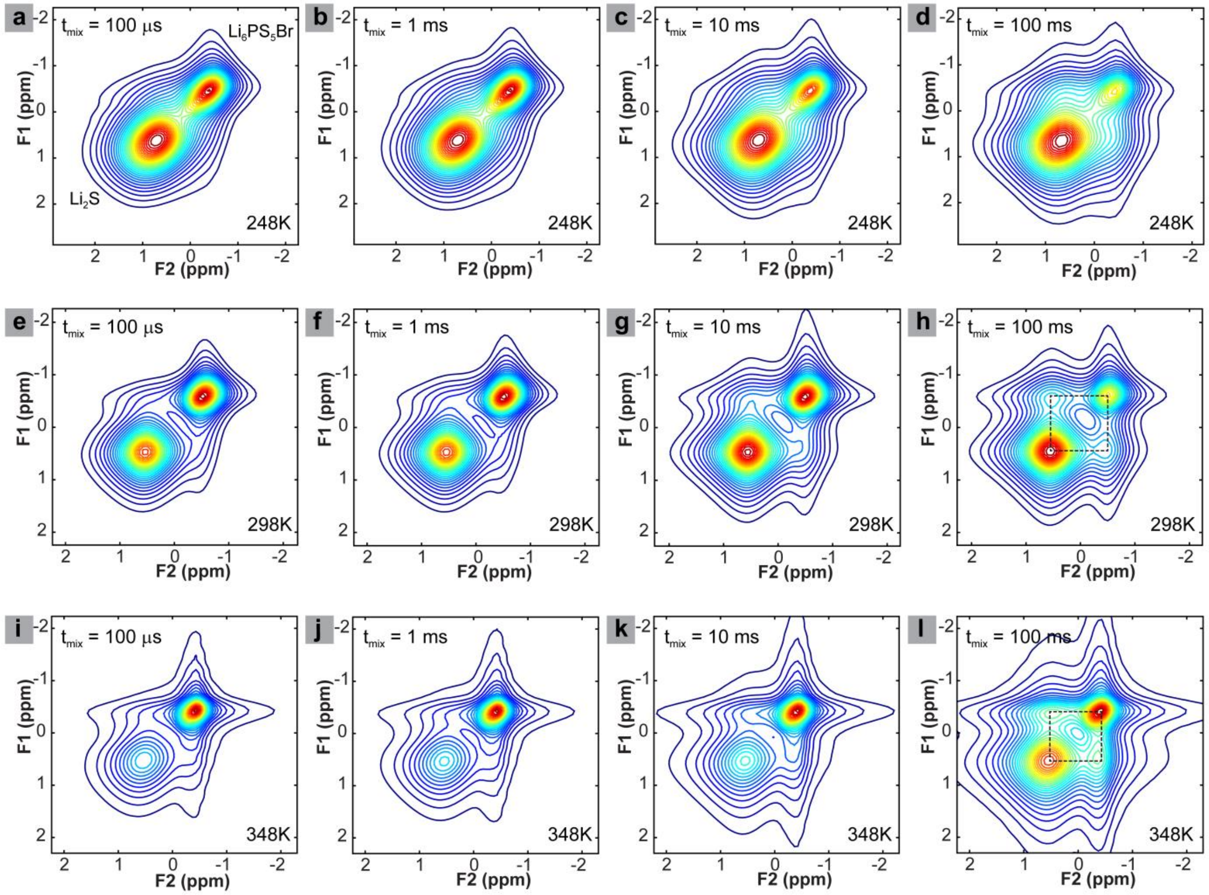

Supplementary Figure 5. NMR measuring the spontaneous lithium-ion transport between the Li_6PS_5Br solid electrolyte and the Li_2S cathode at various temperatures. Two-dimensional ^{7}Li – ^{7}Li exchange spectra (2D-EXSY) measured under magic angle spinning (MAS) at ^{7}Li resonance frequency of 155.506 MHz and spinning speed of 20 kHz for the Li_6PS_5Br-Li_2S electrolyte-electrode mixture III at (a)-(d) 248 K, (e)-(f) 298 K, and (i)-(l) 348 K at mixing times of (a)/(e)/(i) 100 μs, (b)/(f)/(j) 1 ms, (c)/(g)/(k) 10 ms, and (d)/(h)/(l) 100 ms respectively. The diagonal signal corresponds to Li_2S and Li_6PS_5Br and the off-diagonal cross-peaks correspond to exchanged Li.

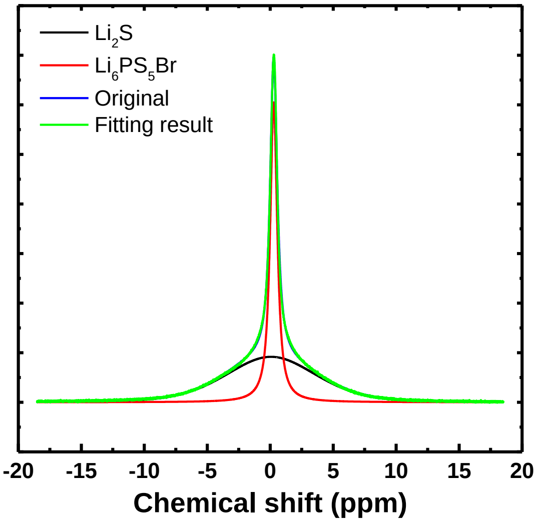

Supplementary Figure 6. NMR line shape of the mixture of Li_6PS_5Br and Li_2S. Static ^{7}Li spectrum of mixture III measured at 298K and a resonance frequency of 155.506 MHz.

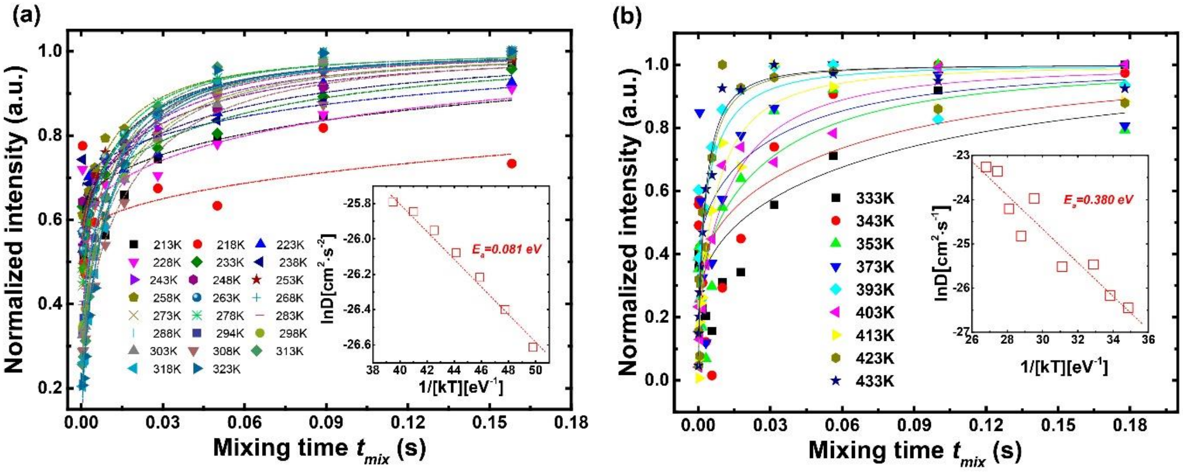

Supplementary Figure 7. 1D exchange measured between Li_6PS_5Br and Li_2S at various temperatures. Normalized intensity of the static Li_2S ^7Li NMR spectrum of (a) pristine and (b) cycled mixture III, T_1 corrected, as a function of mixing time at different temperatures. The inset shows the temperature dependence of the diffusion parameter D, derived from the fits representing the diffusion model (subsequent text). An Arrhenius law is used to fit the activation energy, E_a, representing the diffusion process over the boundary between the Li_6PS_5Br solid electrolyte and the Li_2S phases.

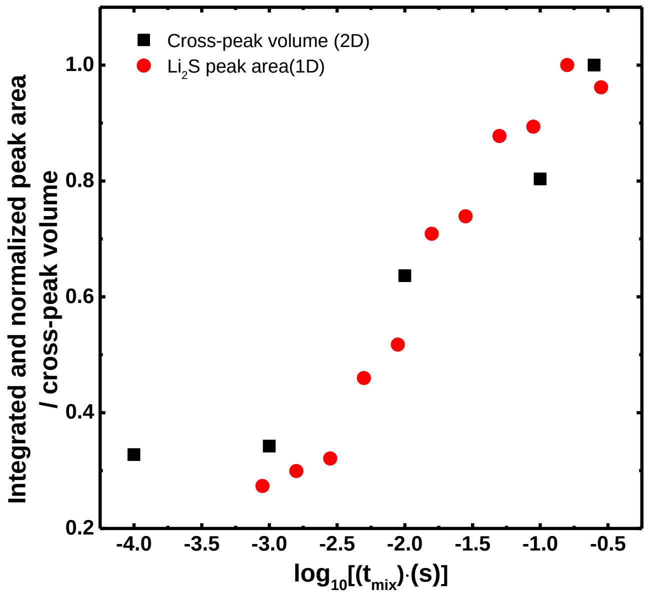

Supplementary Figure 8. Comparison of 1D- and 2D- exchange NMR. Evolution of cross peak volume from the 2D exchange spectra for the pristine mixture III, and the area of the remerging Li_2S signal from the 1D exchange spectra, both as a function of mixing time, at 298K.

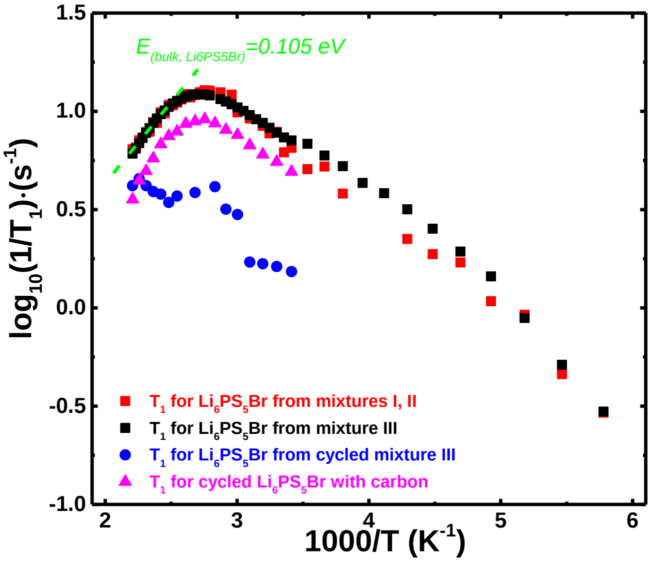

Supplementary Figure 9. Comparison of spin-lattice relaxation rates. Temperature dependence of the ^{7}Li spin-lattice relaxation NMR rates T_1^{-1} measured for Li_6PS_5Br in mixtures I-III and in the cycled mixture III.

Supplementary Table

Supplementary Table 1. EIS fitting results based on various equivalent circuits*

R(RQ)Q					
		R_1/Ω	Error/%	R_2/Ω	Error/%
Mixture I	fresh	137.2	2.728	23	23.08
	cycled	170.0	10.81	-	-
Mixture II	fresh	137.8	0.6	7.6	14
	cycled	122.8	148.1	244.2	90.4
Mixture III	fresh	111.1	0.3	9.2	9
	cycled	139.5	9.54	560.8	4.85
Mixture IV	fresh	250.5	0.7	24.4	46
	cycled	370.1	0.9	130	78

R(RQ)(RQ)Q							
		R_1/Ω	Error/%	R_2/Ω	Error/%	$R_3//\Omega$	Error/%
Mixture I	fresh	-	-	-	-	-	-
	cycled	177.1	7.11	89.65	17	110	10.3
Mixture II	fresh	141.3	1.31	540.8	166.1	-	-
	cycled	190.4	5.4	87.2	18	44.1	22
Mixture III	fresh	111.0	1.47	-	-	-	-
	cycled	129.2	3.6	633.5	1.6	62.8	20
Mixture IV	fresh	251.4	2.92	40.08	72.84	-	-
	cycled	373.7	0.71	127.6	33.3	-	-

R(RQ)(Q(RW))							
		R_1/Ω	Error/%	R_2/Ω	Error/%	R_3/Ω	Error/%
Mixture I	fresh	-	-	-	-	-	-
	cycled	-	-	-	-	-	-
Mixture II	fresh	141.3	1.31	-	-	-	-
	cycled	59.96	-	9341	-	-	-
Mixture III	fresh	111	1.468	-	-	-	-
	cycled	-	-	212.1	-	1824	5.157
Mixture IV	fresh	251.4	2.918	40.08	72.84	-	-
	cycled	373.7	0.7146	127.6	33.3	-	-

***** The values of R_1 and R_2 marked in green are the most accurate fits, as described in the supplementary notes.

Supplementary Notes

Fitting of EIS data

Resistances resulting from the EIS fits in Supplementary Figure 4, using three equivalent circuit models for mixtures I-IV. The in R_1 reflects the resistance of the solid electrolyte, and R_2 reflects the Li_2S cathode electrode-Li_6PS_5Br solid electrolyte interface. The application of the two other equivalent circuits $R_1(R_2Q_2)(R_3Q_3)Q_4$ is motivated by the appearance of a new semicircle in the cycled data, indicating the formation of an additional interface upon cycling, possibly at the In anode. The values of R_1 and R_2 marked in green (Supplementary Table 1) are the most accurate fits, resulting from the fit shown in Supplementary Figure 4. Part II. When values are absent, the error in the resistance is more than 100%.

Quantification of interfacial exchange

Quantification of exchange between the Li_2S and Li_6PS_5Br species was performed by fitting the growing Li_2S signal to a diffusion model wherein a solution to Fick's law for diffusion is determined

It is given by $\frac{\partial m(\vec{r},t)}{\partial t} = \vec{\nabla}^2 \cdot \{D(\vec{r})m(\vec{r},t)\}$, where $m(\vec{r},t)$ is the magnetization of Li at position $\vec{r}$ and *t*, and *D* is the Li-ion self-diffusion coefficient. We assume that the equilibrium exchange between the electrode and electrolyte can be described by an effective diffusion coefficient. Using the mathematical model for spin-diffusion described by Schmidt-Rohr *et al.*[1] the rate of magnetization of the electrode particles is described by the difference between the initial magnetization and the rate of demagnetization of the electrolyte particles. Making the assumption that the Li_6PS_5Br solid electrolyte particles are cube shaped and are embedded in an infinite Li_2S matrix, the analytical expression for the rate of magnetization of the Li_2S phase from the Li_6PS_5Br phase can be given as[2]

$$m(\mathrm{t}_{\mathrm{mix}}) = 1 - \left\{ \frac{m_0}{2}\sqrt{4D\mathrm{t}_{\mathrm{mix}}}\left[ierfc\left(\frac{d}{\sqrt{4D\mathrm{t}_{\mathrm{mix}}}}\right) + ierfc\left(\frac{-d}{\sqrt{4D\mathrm{t}_{\mathrm{mix}}}}\right) - \frac{2}{\sqrt{\pi}} \right] \right\}^3 \quad (1)$$

Where $ierfc(x) = \frac{1}{\sqrt{\pi}} exp(-x^2) - x(1-erf(x))$ and d the Li diffusion distance from the Li_6PS_5Br particle to Li_2S. We assume that diffusion occurs from the center of a Li_6PS_5Br solid electrolyte particle to the center of a Li_2S particle for the sake of simplicity. The average particle sizes are determined from the FWHM of their XRD patterns to be 23 nm and 38 nm for the Li_6PS_5Br and Li_2S phases respectively from mixture III, and due to the larger Li_2S crystallite size and much lower conductivity[3,4] our assumption of an infinite Li_2S is reasonable. The average diffusion distance from the center of a Li_6PS_5Br particle to a Li_2S one is therefore approximately d = 30 nm. It should be realized that the resulting diffusion coefficient is directly correlated to the lithium diffusion distance *d,* and the assumption that the Li-diffusion occurs from the center of a Li_6PS_5Br particle to the center of the Li_2S particle is most likely an overestimation due to the poor conductivity of the Li_2S grains, thereby leading to an overestimation of the diffusion coefficient. To compare the Li-ion dynamics in Li_6PS_5Br obtained using different methods with the conductivity of Li_6PS_5Br measured at 298 K using impedance spectrometry, this obtained jump rate was roughly converted into Li-ion conductivity using both the Nernst-Einstein equation and the Einstein-Smoluchowski equation.[5,6] A comparison of the various Li-ion conductivities determined is given in Figure 4.

Supplementary References

1 Schmidt-Rohr, K. & Spiess, H. W. *Multidimensional Solid-State NMR and Polymers*. (Academic Press, 1994).

2 Wagemaker, M., Kentgens, A. & Mulder, F. Equilibrium lithium transport between nanocrystalline phases in intercalated TiO_2 anatase. *Nature* **418**, 397-399 (2002).

3 Mousa, M., Oei, Y. & Richtering, H. NMR investigations of cation diffusion in some solids with antifluorite structure. *J. Phys. Colloq.* **41**, C6-223-C226-226 (1980).

4 Yang, Y. *et al.* High-capacity micrometer-sized Li_2S particles as cathode materials for advanced rechargeable lithium-ion batteries. *J. Am. Chem. Soc.* **134**, 15387-15394 (2012).

5 Kuhn, A. *et al.* Li ion dynamics in Al-doped garnet-type $Li_7La_3Zr_2O_{12}$ crystallizing with cubic symmetry. *Z. Phys. Chem.* **226**, 525-537 (2012).

6 Buschmann, H. *et al.* Structure and dynamics of the fast lithium ion conductor "$Li_7La_3Zr_2O_{12}$". *Phys. Chem. Chem. Phys.* **13**, 19378-19392 (2011).
